# Establishment of basal cell carcinoma animal model in Chinese tree shrew (*Tupaia belangeri chinensis*)

**DOI:** 10.24272/j.issn.2095-8137.2017.045

**Published:** 2017-07-18

**Authors:** Li-Ping Jiang, Qiu-Shuo Shen, Cui-Ping Yang, Yong-Bin Chen

**Affiliations:** ^1^Key Laboratory of Animal Models and Human Disease Mechanisms, Kunming Institute of Zoology, Chinese Academy of Sciences, Kunming Yunnan 650223, China; ^2^Kunming College of Life Science, University of Chinese Academy of Sciences, Kunming Yunnan 650204, China

**Keywords:** Chinese tree shrew, Basal cell carcinoma, Hedgehog

## Abstract

Basal cell carcinoma (BCC) is the most common skin cancer worldwide, with incidence rates continuing to increase. Ultraviolet radiation is the major environmental risk factor and dysregulation of the Hedgehog (Hh) signaling pathway has been identified in most BCCs. The treatment of locally advanced and metastatic BBCs is still a challenge and requires a better animal model than the widely used rodents for drug development and testing. Chinese tree shrews (*Tupaia belangeri chinensis*) are closely related to primates, bearing many physiological and biochemical advantages over rodents for characterizing human diseases. Here, we successfully established a Chinese tree shrew BCC model by infecting tail skins with lentiviral SmoA1, an active form of Smoothened (Smo) used to constitutively activate the Hh signaling pathway. The pathological characteristics were verified by immunohistochemical analysis. Interestingly, BCC progress was greatly enhanced by the combined usage of lentiviral SmoA1 and shRNA targeting Chinese tree shrew *p53*. This work provides a useful animal model for further BCC studies and future drug discoveries.

## INTRODUCTION

Basal cell carcinoma (BCC) is the most common non-melanoma skin cancer (NMSC), accounting for over 80% of NMSC cases (Rubin et al., 2005). Exposure to ultraviolet radiation is the greatest oncogenic factor for this disease. Most BCCs occur in superficial sites, including the head, neck, trunk, and extremities (Bastiaens et al., 1998; Scrivener et al., 2002), whereas some sites, such as the axillae, breasts, perianal area, genitalia, palms, and soles, are readily ignored by dermatologists during medical examinations (de Giorgi et al., 2005; Rubin et al., 2005). Generally, human skin under both sun and non-sun exposure has the capability to form cancer, indicating that BCC formation could be a multifactor-induced oncogenic process with other genetic factors involved (De Giorgi et al., 2006). The most common histological BCC subtypes are nodular BCCs, followed by superficial BCCs and infiltrative BCCs (Bastiaens et al., 1998; Betti et al., 2012; Scrivener et al., 2002). 

The incidence of BCC continues to increase worldwide (Lomas et al., 2012). Due to different standards, however, it is difficult to compare incidences among countries. Currently, Europe, North America, and Australia top the global incidence rates. For example, the rates have increased approximately 5% every year over recent decades in Europe (Lomas et al., 2012), and cases in the USA now exceed 2.8 millon patients, outnumbering the total rates of all other cancers (Asgari et al., 2015; Rogers et al., 2015; Siegel et al., 2016) and accounting for 3 000 deaths annually (Madan, 2010; Mohan & Chang, 2014). Although the incidence of BCC obviously increases with age, the incidence in adults younger than 40 has also increased year by year (Christenson et al., 2005; Demers et al., 2005). Currently, it costs the government more than $40 million (USD) to provide medical care annually in USA (Chen et al., 2001; Mudigonda et al., 2010). In Australia, one in two people by the age of 70 will be diagnosed with BCC (Lomas et al., 2012; Staples et al., 2006). In Africa and South America, the rates also have increased but relatively slowly (Abarca & Casiccia, 2002; Rawashdeh & Matalka, 2004). 

The Hedgehog (Hh) signaling pathway is an evolutionarily conserved pathway known to play essential roles in embryonic development and adult tissue hemostasis and repair (Chen & Jiang, 2013). In general, the Hh ligand is bound to the secreted twelve-transmembrane receptor Patched-1 (Ptch1). Smoothened (Smo), a seven transmembrane receptor, is then activated by phosphorylation and other post-translational modifications, leading to accumulation in the primary cilium and induction of the Gli transcription factor to activate downstream gene expression (Yang et al., 2012). Malfunction of the Hh signaling pathway results in various developmental defects, including holoprosencephaly, cyclopia, limb abnormalities, and progression of tumors such as BCC and medulloblastoma (Teglund & Toftgård, 2010). Studies have demonstrated some Hh signaling component mutations associated with BCC development (De Zwaan & Haass, 2010; Lacour, 2002; Reifenberger et al., 2005). However, the predominant oncogenic mutations are those of the *Ptch1* and *Smo* genes, which can cause abnormal constitutive activation of the Hh signaling pathway (Bonilla et al., 2016; Sekulic & Von Hoff, 2016; Xie et al., 1998). Although activation of the Gli transcriptional factor sequestered by Sufu protein loss of function should promote BCC progression, inactivated Sufu in mouse skin shows few or no BCCs (Li et al., 2014), suggesting that Ptch1 or Smo might be a better target for establishing a BCC animal model. 

There are many therapies for the treatment of BCC in cancer patients, including the modulation of Hh signaling activities for invasive BCC (Sekulic et al., 2012; Tang et al., 2012; Von Hoff et al., 2009). Vismodegib (GDC-0449), a Smo specific antagonist approved by the Food and Drug Administration (FDA) in 2012, is used to treat metastatic or locally advanced BCCs (Dlugosz et al., 2012). A recent study showed that an amino acid substitution at a conserved specific aspartic acid residue of a *SMO* mutation could confer BCC patients resistance to GDC-0449 treatment, suggesting that targeting *SMO* might be important for BCC treatment. As such, exploration of second-generation *SMO* inhibitors that are capable of overcoming acquired resistance is increasing (Yauch et al., 2009). Sonidegib (LDE225), another Smo antagonist approved by the FDA in 2015, is a clinical drug used for locally advanced BCC (Burness, 2015). It has also been reported that the antifungal drug itraconazole can suppress all known Smo drug-resistant mutants, thus inhibiting the Hh signaling pathway (Kim et al., 2013). 

To further explore BCC pathogenesis, as well as develop new strategies for treating BCC, better animal models are required. Such models should conform to the conditions of patients and allow for: (1) the time of BCC induction to be defined and controllable; (2) the development of various stages and subgroups of human BCC; and (3) the inductivity of BCC in 100% of animals (Chen et al., 2009). To meet these requirements, many BCC models have been established. Most are transgenic mouse models, such as *Ptch1* knockout mice (Arad et al., 2008; Aszterbaum et al., 1999; Nitzki et al., 2010; Skvara et al., 2011; So et al., 2004), or include constitutive activation of other Hh signaling pathway key regulators, such as oncogenic *Smo*, *Gli1*, or *Gli2* mutation expressions driven by skin-specific keratin (K) 5, 6, or 14 promoters (Nitzki et al., 2012). In seven-week-old Sprague-Dawley rats, e.g., spontaneous BCC tumors were observed as single, reddish-brown subcutaneous masses located at the left inguinal region, basaloid cells showed lobular and cribriform growth with high mitotic rates, and cytokeratin 14 and cytokeratin 18 were expressed in nest tumor cells, thereby indicating that spontaneous BCC can occur in young rats (Lee et al., 2010). Nano-electro-ablation methods have been found to induce apoptosis efficiently in a Ptch1 (+/-) K14-Cre-ER *p53* fl/fl mouse BCC model (Nuccitelli et al., 2012). Protein kinase A (PKA) activation by cAMP agonist forskolin inhibited BCC growth, particularly drug resistant BCC for Smo inhibitors, which was performed and evaluated in tamoxifen-induced 30-day-old postnatal mice which were born from male K14-CreERT2 crossed with female homozygous R26-SmoM2 (Makinodan & Marneros, 2012). Furthermore, introduction of Smoothened constitutive active form SmoA1 in mouse cerebellar granule neuron precursors was shown to cause a 48% incidence rate of medulloblastoma (Hallahan et al., 2004). 

Inactivation of tumor suppressor *p53* promotes tumorigenesis and is correlated with poor survival (Ghaderi & Haghighi, 2005; Lacour, 2002; Moles et al., 1993; Urano et al., 1995; Wang et al., 2017; Ziegler et al., 1993). Thus, the clues to the mutation of *p53* in human BCCs show that their ablation might also contribute to tumor formation (Wörmann et al., 2016; Wu et al., 2014). Therefore, to imitate spontaneous BCCs in humans and speed up progression in animals, disruption of *p53* could be an alternative. 

Considering the distant relationship between humans and rodents, and the long period for non-human primate model establishment, we choose the Chinese tree shrew (*Tupaia belangeri chinensis*) as an animal model for BCC. The Chinese tree shrew, which belongs to Tupaiidae (Scandentia), is widely spread over Southeast Asia and Southwest China, including Yunnan province (Zhao et al., 2014). This tree shrew species possesses a variety of unique and notable physiological characteristics, including small adult body size, high brain-to-body mass ratio, short reproductive cycle and life span, low maintenance, and most importantly, a close affinity to primates (Fan et al., 2013). The recent elucidation of the genome of *Tupaia belangeri chinensis* confirmed the close genomic relationship between *Tupaia belangeri* and primates (Fan et al., 2013). As a favorable animal model, the tree shrew has been used for many human disease studies, including research on depression (Fuchs, 2005; Wang et al., 2011; 2012; 2013), drug addiction (Sun et al., 2012; Zhang et al., 2011), virus infection (Amako et al., 2010; Yan et al., 1996; Yang et al., 2005), bacterial infection (Li et al., 2012), breast cancer (Elliot et al., 1966; Ge et al., 2016; He et al., 2016; Xia et al., 2012), glioblastoma (Tong et al., 2017), thrombosis (Endo et al., 1997), metabolic diseases (Wu et al., 2013; 2016; Zhang et al., 2015; 2016), stem spermatogonium transgenics (Li et al., 2017), and myopia (Norton et al., 2006). Recently, pharmacological research through drug target prediction and genomic and transcriptomic scale analysis has shown that more than half of the drug target proteins identified from the tree shrew genome demonstrate higher similarity to human targets than that of the mouse, as validated by the constitutive expression of proteinase-activated receptors (Zhao et al., 2014). The above studies indicate that over several years of research, the tree shrew has shown huge potential as an animal model for research of human diseases, including mental, nervous, infective, metabolic, and cancer diseases (Xiao et al., 2017; Xu et al., 2013; Yao, 2017), as well as drug safety (Zhao et al., 2014). 

To establish a BCC model in the tree shrew, we constructed lentiviral vectors containing Hh signaling pathway constitutive activator SmoA1 tagged by GFP, which was used to trace the lentiviral infected tree shrew skin cells. We then infected the dorsal skins of 6-week-old tree shrews *in vivo* with both control and SmoA1 containing lentiviruses using one dose (10μL) of the virus containing 5.6×10^5^ transducing units (TU). Two weeks later, hematoxylin-eosin (HE) staining was performed to examine the pathological phenotypes of the skins. The results showed the human BCC-like phenotype and remarkable pathological changes compared with reciprocal biopsies from the control virus. Interestingly, when we injected the virus into the tree shrew tail skins, the BCC tumor formed more easily than that on other parts of skin after only one dose containing 5μL of pCDH-SmoA1 virus (5.6×10^5^ TU) and 5μL of lentiviral shRNA targeting *p53* (2×10^5^ TU). In summary, we successfully and efficiently established a BCC model using the tree shrew, which closely recapitulated the clinical phenomena. This animal model will help to better understand the fundamental mechanisms of BCC, and could be used for evaluating novel therapeutic strategies against BCC and pre-clinical drugs in the future. 

## MATERIALS AND METHODS

### Animal use and care

Wild-type adult male tree shrews were provided by the Kunming Primate Research Center, Kunming Institute of Zoology (KIZ), Chinese Academy of Sciences (CAS). All experimental procedures and animal care and handling were performed under the standard guidelines approved by the Institutional Animal Care and Use Committee of the KIZ, CAS (SMKX2013023). 

### Plasmids construction and cell culture

A mSmoA1-6×myc fragment was collected from pGE-*mSmoA1* digested by double restriction endonuclease with *Hind* *Ⅲ* and *Sac II*, and was then cloned into pCDH empty expression vector digested by *EcoR I* and *BamH I* in blunting form. The short heparin RNA (shRNA) targeting sequences for tree shrew *p53* (*tsp53*) were 1^#^: 5'-CCTCAGCATCTTATCCGGGTG-3' and 2^#^: 5'-TTTGTGCCTGTCCTGGAAGAG-3', and the control scramble shRNA sequence was 5'-GCACTACCAGAGCTAACTCAG-3'. The shRNA oligos were synthesized by BGI-Shenzhen (Shenzhen, China). The synthesized complementary oligo DNA was annealed by 95 ℃ boiling water and ligated with pLKO.1 plasmid. The product was transformed into DH5± competent cells and plated on LB agar. Individual colonies were randomly collected and shaken at 37 ℃, with the plasmids then extracted using a plasmid extraction kit (Tiangen, Beijing, China) and checked by enzyme digestion and sequencing. Primary culture of tree shrew skin derived progenitor/stem cells (SKPs) was performed according to a previously validated method (Biernaskie et al., 2007). Briefly, animals were euthanized by ethyl ether anesthesia and dissected for the generation of dorsal back skin and tail skin. All blood vessels, adipose, fascia, and muscle underlying the dermis were removed gently to reduce contamination by other cell types in the culture. The dissected skin tissues were minced into 1-2 mm^2^ size pieces, transferred to a 15 mL conical tube for digestion, and submerged in 0.1% trypsin for 15-60 min at 37 ℃. Afterwards, 10 mL of Dulbecco modified Eagle's medium (DMEM/F12) (Hyclone) supplemented with 10% fetal bovine serum (FBS) (Hyclone) was added to stop the trypsin digestion process. The samples were then centrifuged at 1 200 r/min and 4 ℃ for 6-8 min and resuspended in 1 mL of DMEM/F12 medium, and filtered through a 40 μm cell strainer. The flow-through samples were cultured continuously as SKP cells. 

The HEK-293T cells were obtained from American type culture collection (ATCC, CAT#: CRL-3216) and cultured in DMEM high glucose (Hyclone), 10% FBS (Hyclone), 1% penicillin (Beyotime Biotechnology, China) and 1% streptomycin (Beyotime Biotechnology, China) in a 37 ℃ and 5% CO_2_ incubator. 

### Lentiviral package and preparation

The lentiviruses were generated according to the manufacturera's protocols (Addgene, USA), with the viruses harvested at 48 h and 72 h after transfection and filtered with a 0.45 μm filter. The tree shrew SKPs were then infected with the viruses or the virus particles were concentrated by ultracentrifugation at 8 000 r/min and 4 ℃ for 3 h before *in vivo* infection. Polybrene (Sigma, USA) (final concentration 4 μg/mL) was added when the tree shrew SKPs were infected to promote infection efficiency as well as *in vivo* infection. Infected SKPs were screened with puromycin (Invitrogen, USA) after 72 h of infection, followed by cell amplification and identification. 

### Real-time quantitative PCR (qPCR) 

The efficiency of ts*p53* (ts: tree shrew) shRNA was tested in tree shrew SKPs. Total RNA was isolated using Trizol reagent (Takara, Japan) and reverse transcription was performed using an iScript cDNA Synthesis Kit according to the manufacturera's instructions (Bio-Rad, USA). This was followed by quantitative real-time PCR using a SYBR Green Mix with Rox (Roche, USA). The primer sequences used were: tsGAPDH: 5'--ACGACCCCT TCATTGACTTG-3'and 5'-TCTCCATGGTGGTGAAGACA-3'; tsP53: 5'-CCACGGAAGACTGGTTCAAT-3' and 5'-ACGTGCAGGTGA CAGACTTG-3'. 

### Lentiviral injection

After ketamine anesthetic (40 μg/g), the hair on the dorsum and tail of the tree shrews was shaved, with depilatory paste then applied to remove fine hair. Next, the pCDH-mSmoA1 lentivirus (5.6×10^5^ TU), shRNA targeting tree shrew *p53* gene lentivirus (shp53, 2×10^5^ TU), and control vector (pCDH-mSmoA1 group, pCDH-mSmoA1 and shp53 group, and control group, respectively) were injected into a certain region of the dorsum and tail. At least 30 domesticated tree shrews (~6-weeks-old) were used. Both normal skin tissues and skin tumors were isolated and collected after animals were sacrificed at two weeks or two months on the dorsum and tail of the tree shrews, respectively. All tissues were fixed for immunohistochemical analysis or immediately frozen by liquid nitrogen and stored at -80 ℃. 

### HE staining

Normal skin tissues and tumors were preserved in 10% phosphate- buffered formalin. Tissues were then processed for paraffin embedding and cut into 4 μm thick sections. Section samples were subjected to standard hematoxylin and eosin (HE) staining. 

### Statistical analysis

All data were presented as means±*SE* of a minimum of three replicates. For all analyses, we evaluated statistical differences using the Student's *t*-test. Each experiment was performed at least three times. Differences were considered significant if the *P* value was < 0.05 (^*^: *P* < 0.05, ^**^: *P* < 0.01, ^***^: *P* < 0.001), compared with the control group. 

## RESULTS

**Figure 1:**
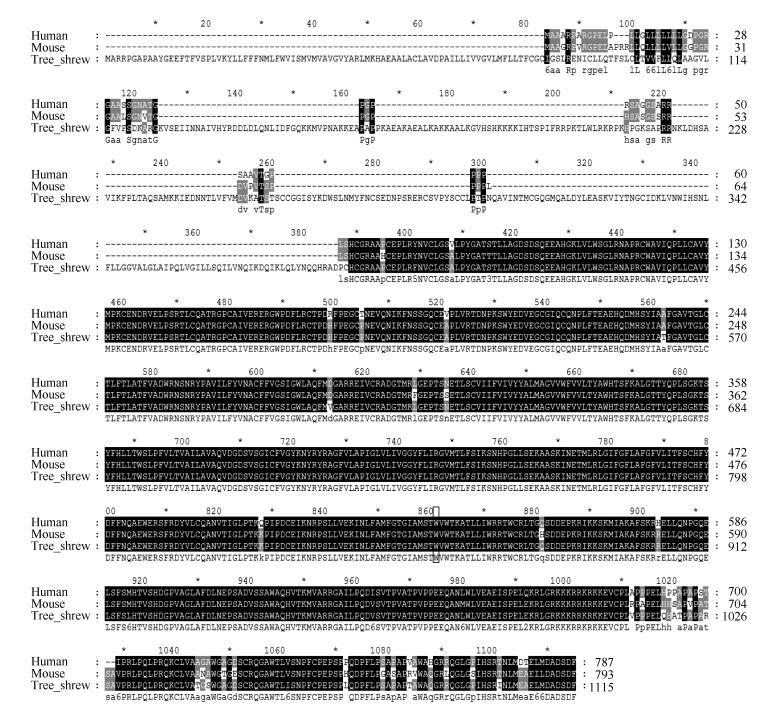
Comparison of human, tree shrew, and mouse Smoothened proteins

**Figure 2:**
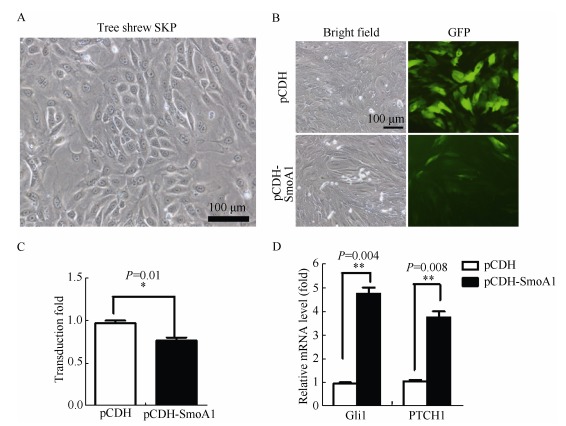
Lentivirus SmoA1 efficiently infected tree shrew SKPs

We performed protein sequence alignment for the Smo protein among humans, tree shrews, and mice using Blast software (<uritalic>https://blast.ncbi.nlm.nih.gov/Blast.cgi</uritalic>). The results showed that the core transmembrane domains as well as the C-terminal of the human, mouse, and tree shrew Smo proteins were highly conserved, although the tree shrew Smo also contained an elongated N-terminal overhang, whose structural and functional roles need to be further validated. While we found that the oncogenic <italic>SmoA1</italic> mutation site (W539 in mice and W535 in humans) was highly conserved among all three species, as shown in <xref ref-type="fig" rid="F1-ZoolRes-38-4-180">Figure 1</xref> (<xref ref-type="bibr" rid="b76-ZoolRes-38-4-180">Taipale et al., 2000</xref>; <xref ref-type="bibr" rid="b93-ZoolRes-38-4-180">Xie et al., 1998</xref>), we decided to induce BCC in tree shrew skins with the constitutive active form of SmoA1 for the following experiments (<xref ref-type="bibr" rid="b13-ZoolRes-38-4-180">Chen et al., 2011</xref>). To validate the lentiviral titer and efficiency for tree shrew skin, we infected tree shrew SKPs with the viruses <italic>in vitro</italic>. As SmoA1 was tagged by green fluorescence protien (GFP), the green fluorescence percentage observed by the fluorescence microscope was used to validate infection efficiency (<xref ref-type="fig" rid="F2-ZoolRes-38-4-180">Figure 2A</xref>-<xref ref-type="fig" rid="F2-ZoolRes-38-4-180">C</xref>). The fluorescence analysis results showed that the SmoA1 lentivirus infected the tree shrew SKPs efficiently by more than 70% (<xref ref-type="fig" rid="F2-ZoolRes-38-4-180">Figure 2C</xref>). We also analyzed the Hh signaling pathway activity after lentiviral <italic>SmoA1</italic> expression, and found the <italic>Ptch1</italic> and <italic>Gli1</italic> mRNA expressions were up-regulated (<xref ref-type="fig" rid="F2-ZoolRes-38-4-180">Figure 2D</xref>). 

**Figure 3:**
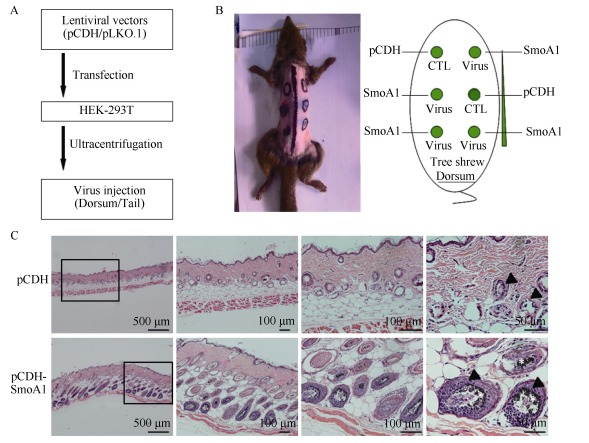
Over-expression of SmoA1 induced BCC-like hyperplasia in dorsal skins of the tree shrews *in vivo*

**Figure 4:**
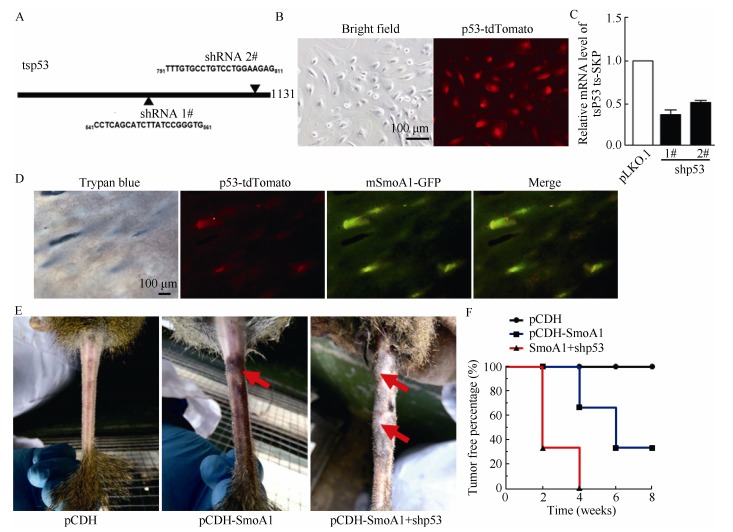
BCC formation in tree shrew tail skins by combined usage of SmoA1 and shp53 viruses

The intracutaneous lentiviral injected dorsal areas of the tree shrew skin are shown in Figure 3B. All tree shrews were intracutaneously injected with 5.6×10^5^ TU virus/injection site with either the control, SmoA1, or *p53* shRNA lentiviruses, respectively, or in combination. No significant weight lost was observed in the animals (data not shown). The total viral mixture volume was approximately 10μL. Two weeks later, the pathologies of the lentivirus infected dorsal skins were analyzed by HE staining. We found that the pCDH-SmoA1 group exhibited human BCC-like pathological characteristics, such as hyperplasia of skin cells with hair follicle (HF) disruption, and pigmentation and nuclear explosion expansion (Figure 3C). However, black plaque did not develop into human-like BCC, even after a longer period. Since *p53* ablation has been frequently observed in BCC and other tumors, which could possibly speed up the process of BCC (Rady et al., 1992; Soussi & Béroud, 2001; Soussi et al., 2000; Wijnhoven et al., 2005), we constructed lentiviral expressing shRNAs targeting tree shrew *p53* with tdTomato expression driven by an individual PGK promoter, which was used to follow the shRNA expressing cells and tissues (Figure 4A). Fluorescence microscopy showed that positive red fluorescence approached 100% in tree shrew SKPs after lentiviral shp53-tdTomato infection (Figure 4B), and tree shrew *Tp53* mRNA knockdown efficiency by lentiviral shRNAs was confirmed by real-time PCR compared with scramble shRNA control (Figure 4C). 

It has been documented previously that the vast majority of BCCs in a conditional mouse model (*K5*-*tTA*;*TRE*-*Gli2* bitransgenic mice) formed on the mice tails, ears, extremities, and dorsal skin (Hutchin et al., 2005). We tested BCC formation efficiency in the tail skins of tree shrews using the above lentiviruses. The results (Figure 4D) indicated that both SmoA1-GFP and shp53-tdTomato successfully infected the tree shrew tail skin. Furthermore, obvious BCC plaque and mass formation were found in the pCDH-SmoA1 group two months later, and the pCDH-mSmoA1 and shp53 groups showed the most malignancies. Statistically, ~40% of tree shrews showed BCC-like phenotypes after four weeks following the SmoA1 viral-injection alone, and reached 60% after 6-8 weeks. Interestingly, more than 70% of tree shrews showed BCC-like phenotypes two weeks after SmoA1 and *p53*-shRNA viral-injection, which reached to 100% after four weeks (Figure 4F). These data suggest that the Hh signaling pathway constitutively activated by SmoA1 overexpression induced tree shrew BCC pathogenesis, and knockdown of tumor suppressor *p53* could accelerate tree shrew BCC tumor progression. 

## DISCUSSION

Vismodegib has been used recently for metastatic or advanced BCC in clinical trials, showing good effect in phase I trials (Graham et al., 2011; Lorusso et al., 2011; Von Hoff et al., 2009), but only 30% of metastatic and 43% of locally advanced BCC patients treated with vismodegib have demonstrated a good response in Phase II trials (Sekulic et al., 2012). In Phase I study of sonidegib, 37% of BCC patients achieved partial or complete response, whereas 42% of BCC patients in Phase 2 responded well to treatment with 200 mg of sonidegib per day orally (Migden et al., 2015). Collectively, these studies suggest that downstream inhibitors of Hh signaling and a combination of therapies targeting other pathways using better animal models are required. Here, we showed that lentiviral injection of SmoA1 and shp53 could induce BCCs in tree shrew skins successfully. 

In addition, a recent study indicated that the MK-4101 molecule can attenuate the Hh signaling pathway through inhibition of Gli, alteration of IGF, and Wnt signaling pathway activities, thus proving to be a promising therapeutic drug for BCC patients (Filocamo et al., 2016). A second-generation antifungal drug posaconazole, which showed distinct mechanisms from cyclopamine or cyclopamine competitive inhibitors, exhibits better drug-drug interaction and fewer side effects than current SMO inhibitors, and could provide a novel strategy for clinical drug combinational therapy (Chen et al., 2016). 

In general, older adults suffering from BCC receive Hh signaling pathway inhibitor treatment. Premenopausal women are also subjected to menopause due to the reversal of chorionic hormone receptor inhibition (Simone et al., 2016). Furthermore, many dermatologists are not familiar with the side effects of such treatment, thus consulting professional practitioners about teratogenicity and sequelae of ovarian failure is required. New or persistent ulcers, nodules, or erythema after three months of treatment with Hh signaling pathway inhibitors have been found by biopsy (Simone et al., 2016; Zhu et al., 2014), therefore all skin should be monitored during the whole treatment process (Simone et al., 2016; Zhu et al., 2014). At the same time, non-BCC damage should also be given comprehensive treatment because synchronous occult amelanotic melanoma has been reported in 25% (3/12) of BCC patients (Simone et al., 2016; Zhu et al., 2014). Two BCC patients, five years after Hh signaling pathway inhibitor treatment, have survived by paying close attention to and interfering with various side effects (Jacobsen et al., 2017). 

Recent research, which established EGFP-tagged transgenic tree shrews following spermatogonial stem cell (SSC) transplantation, provided a good approach for the generation of multiple human disease models using the tree shrew by gene editing manipulation (Li et al., 2017). Although BCC was not observed in DMBA/TPA combination treated wild-type mice (Indra et al., 2007), it has been successfully generated in Ptch^flox/flox^CD4Cre^+/-^ mice (Uhmann et al., 2014). Thus, it would be interesting to combine DMBA/TPA with lentiviral SmoA1and p53-shRNA in tree shrew skins in the future. 

PTEN (phosphatase and tensin homolog deleted on chromosome 10) plays critical roles in tissue homeostasis and cancer development, and is a commonly mutated tumor suppressor gene (Salmena et al., 2008). Earlier research showed that 100% of mice with complete *Pten* deficiency in their keratinocytes and a proportion with *Pten* heterozygosity, developed NMSC spontaneously (Suzuki et al., 2003). Deletions of *Pten* in BCC are an infrequent event (Quinn et al., 1994), implicating that *Pten* is a significant suppressor of non-melanoma skin tumorigenesis (Hertzler-Schaefer et al., 2014; Macdonald et al., 2014; Ming & He, 2009). To improve the BCC tree shrew model, loss of PTEN function as well as UV radiation might stimulate low-level Hh signaling caused by mutations in Hh pathway components via the up-regulation of the PI3K/AKT pathway and DNA damage-related signaling activation, respectively ( Ming & He, 2009; Ouhtit et al., 1998). 

BCCs are closely related to abnormal oncogenic activation of the Hh pathway, which can have different functions and mechanisms between different species, the closer relationship between different species, the closer functions and mechanisms of relative genes. These similar biological characteristics between animals and human allow for the mimicry of human tumor progression. However, there are limits to murine animal models. Genome analysis has verified that the tree shrew is closely related to primates (Fan et al., 2013) and is superior to murine species. Small body size, low-cost maintenance, short reproductive cycle and life span, and its close relationship to primates make the tree shrew a safer, more efficient, and more predictable animal model, therefore surpassing murine species in the testing of drug efficacy and safety and deciphering the pathogenesis of BCC. Here we established, for the first time, a tree shrew BCC model that successfully simulated human BCC pathological features. However, the molecular markers of BCCs are needed to confirm this model at the molecular level. It would be interesting to use current clinical BCC-treatment drugs, such as vismodegib, to validate the efficiency and effects of the tree shrew BCC model. Furthermore, this model could be used to screen novel natural compounds that might function alone or in synergy with current clinical drugs to treat BCC.
